# Canagliflozin alleviates valproic acid-induced autism in rat pups: Role of PTEN/PDK/PPAR-γ signaling pathways

**DOI:** 10.3389/fphar.2023.1113966

**Published:** 2023-02-22

**Authors:** Mariam A. Elgamal, Dina M. Khodeer, Basel A. Abdel-Wahab, Ibrahim Abdel Aziz Ibrahim, Abdullah R. Alzahrani, Yasser M. Moustafa, Azza A. Ali, Norhan M. El-Sayed

**Affiliations:** ^1^ Egypt Healthcare Authority, Comprehensive Health Insurance, Port-Said, Egypt; ^2^ Department of Pharmacology and Toxicology, Faculty of Pharmacy, Suez Canal University, Ismailia, Egypt; ^3^ Department of Pharmacology, College of Pharmacy, Najran University, Najran, Saudi Arabia; ^4^ Department of Pharmacology and Toxicology, Faculty of Medicine, Umm Al-Qura University, Makkah, Saudi Arabia; ^5^ Dean of Faculty of Pharmacy, Badr University in Cairo, Badr City, Egypt; ^6^ Department of Pharmacology and Toxicology, Faculty of Pharmacy (Girls), Al-Azhar University, Cairo, Egypt

**Keywords:** valproic acid, canagliflozin, Pten, PPAR γ, PDK, autism

## Abstract

Autism is complex and multifactorial, and is one of the fastest growing neurodevelopmental disorders. Canagliflozin (Cana) is an antidiabetic drug that exhibits neuroprotective properties in various neurodegenerative syndromes. This study investigated the possible protective effect of Cana against the valproic acid (VPA)-induced model of autism. VPA was injected subcutaneously (SC) into rat pups at a dose of 300 mg/kg, twice daily on postnatal day-2 (PD-2) and PD-3, and once on PD-4 to induce an autism-like syndrome. Graded doses of Cana were administered (5 mg/kg, 7.5 mg/kg, and 10 mg/kg, P.O.) starting from the first day of VPA injections and continued for 21 days. At the end of the experiment, behavioral tests and histopathological alterations were assessed. In addition, the gene expression of peroxisome proliferator-activated receptor γ (PPAR γ), lactate dehydrogenase A (LDHA), pyruvate dehydrogenase kinase (PDK), cellular myeloctomatosis (c-Myc) with protein expression of glucose transporter-1 (GLUT-1), phosphatase and tensin homolog (PTEN), and level of acetylcholine (ACh) were determined. Treatment with Cana significantly counteracted histopathological changes in the cerebellum tissues of the brain induced by VPA. Cana (5 mg/kg, 7.5 mg/kg, and 10 mg/kg) improved sociability and social preference, enhanced stereotypic behaviors, and decreased hyperlocomotion activity, in addition to its significant effect on the canonical Wnt/β-catenin pathway *via* the downregulation of gene expression of LDHA (22%, 64%, and 73% in cerebellum tissues with 51%, 60%, and 75% in cerebrum tissues), PDK (27%, 50%, and 67% in cerebellum tissues with 34%, 66%, and 77% in cerebrum tissues), c-Myc (35%, 44%, and 72% in cerebellum tissues with 19%, 58%, and 79% in cerebrum tissues), protein expression of GLUT-1 (32%, 48%, and 49% in cerebellum tissues with 30%, 50%, and 54% in cerebrum tissues), and elevating gene expression of PPAR-γ (2, 3, and 4 folds in cerebellum tissues with 1.5, 3, and 9 folds in cerebrum tissues), protein expression of PTEN (2, 5, and 6 folds in cerebellum tissues with 6, 6, and 10 folds in cerebrum tissues), and increasing the ACh levels (4, 5, and 7 folds) in brain tissues. The current study confirmed the ameliorating effect of Cana against neurochemical and behavioral alterations in the VPA-induced model of autism in rats.

## 1 Introduction

Autism spectrum disorder (ASD), a neurodevelopmental disorder, is broadly detected within the first three years of life (Benger et al., 2018). Autism is characterized by major core behaviors: deficits in sociability either for communication or interaction, repetitive behaviors, interests, and thoughts, with other non-core traits, including self-injury, hyperactivity features, and sensitivity to stimulation (Choi et al., 2018; Dai et al., 2018; Eissa et al., 2018). Autistic patients show many variations in the cerebellum, especially loss of Purkinje cells (el Falougy et al., 2019). Moreover, cerebellum dysfunction disrupts the prefrontal cortex’s function (Forbes and Grafman, 2010).

Non-controlled epileptic attacks during pregnancy produce high risk of injury to both the mother and fetus (Klein, 2011). So, epileptic pregnant women must continue on VPA medication (Stephen et al., 2012). VPA crosses the placenta and accumulates in the fetal circulation with higher concentration than that in the maternal blood, causing toxicity and teratogenicity (Vajda, 2012). VPA exposure during early pregnancy showed classical signs of autism with developmental and behavioral delays (Christensen et al., 2013; Kim et al., 2017).

Current mainstay treatments for ASD are only behavioral treatments against the core symptoms of ASD (Aishworiya et al., 2022). There are no pharmacological treatments that treat the core symptoms of ASD. Some medications seek only to reduce co-occurring symptoms associated with ASD: attention deficit hyperactivity disorder (ADHD), self-harming behavior, anxiety, depression, seizures, sleep problems, gastrointestinal problems, phobias, intellectual disability, and speech/language impairment (Hyman et al., 2020; Aishworiya et al., 2022).

The canonical Wnt/β-catenin pathway mainly participates in central nervous system (CNS) development, especially cognitive disorders (Kwan et al., 2016). Additionally, the canonical pathway is upregulated in ASD (Mulligan and Cheyette, 2016). Upregulation of the Wnt/β-catenin pathway stimulates aerobic glycolysis, the Warburg effect, throughout the activation of the glucose transporter (GLUT), 3-phosphoinositide-dependent kinase 1 (PDK1), and lactate dehydrogenase A (LDHA) (Vallée and Vallée, 2018).

Overstimulation of the Wnt/β-catenin pathway enhances the Wnt/β-catenin target genes’ transduction process; cellular myeloctomatosis (c-Myc) expression (Yue et al., 2010) consecutively leads to further expression of genes encoding the enzymes of aerobic glycolysis: LDHA, PDK, and GLUT (Yang et al., 2012; Vallée and Vallée, 2018). GLUT subtype is crucial for the homeostasis of glucose transport (McEwen and Reagan, 2004). Moreover, activated PDK1 leads to the conversion of pyruvate to lactate through LDHA (Vallée and Vallée, 2018) while blocking its conversion to acetyl-CoA, and finally, it leads to the destruction of acetylcholine (ACh) formation from acetyl-CoA (Roche et al., 2001).

Canagliflozin (Cana) is a sodium-glucose co-transporter type 2 (SGLT2) inhibitor used for type 2 diabetes mellitus management (Naznin et al., 2017). Cana is recognized to have a neuroprotective effect on cisplatin-induced peripheral neurotoxicity in rats (Abdelsameea and Kabil, 2018). Furthermore, Cana has a valuable impact on the scopolamine induction rat model of memory impairment (Wiciński et al., 2020). Similarly, empagliflozin, another SGLT2 inhibitor, remarkably blocked the impaired cognitive function in a type 2 diabetes model in mice (Wiciński et al., 2020). Additionally, high and low doses of empagliflozin inhibited the neurological defects of the ischemia induction model in rats (Wiciński et al., 2020).

Therefore, this study evaluated the protective role of Cana in rat pups against autism induced by VPA focusing on PTEN/PDK/PPAR-γ signaling pathways and their impact on various behaviors as possible mechanisms involved in its neuroprotection.

## 2 Materials and methods

### 2.1 Drugs and chemicals

Sodium salt of VPA was purchased from Sigma-Aldrich (St. Louis, MO, United States). VPA was prepared by dissolving it in normal saline (100 mg/mL) (Favre et al., 2013; Morakotsriwan et al., 2016). Cana was generously granted by Soficopharm Company (Cairo, Egypt). Cana was prepared by dissolving it in distilled water immediately before use. All other chemicals used in the study were of analytical grade and obtained from Adwic Co. (Cairo, Egypt).

### 2.2 Animals

Newborn Sprague–Dawley rat pups were born on postnatal day 0 (PD-0) (Mony et al., 2016; 2018). Among inclusion criteria for pups’ selection was that the mother should give birth to 6–8 pups. Pups were housed with their mothers in stainless steel cages with free access to food and water, room temperature 24°C ± 1°C, and a 12-h light–dark cycle. All animal experiments were approved by the Ethical Committee for the Animal Research of Faculty of Pharmacy, Suez Canal University (approval no. 201911PHDA1). Behavior studies were performed during the daytime between 10.00 a.m. and 4.00 p.m.

### 2.3 Induction of autism in rat pups

Rat pups were subcutaneously (SC) injected in the dorsal neck region with VPA at a dose of 300 mg/kg twice daily on PD-2 and PD-3, and once on PD-4. The control group was SC injected with an equal amount of saline (Lee et al., 2016; Mony et al., 2016).

### 2.4 Experimental design

Pups of each mother (6–8) were randomly distributed over five experimental groups (10 pups each), and they were housed with their mother. Each experimental group was marked with different colors by spots on their back, and the marks were checked every other day. The study included rat pups of both sexes, starting with a ratio of 1:1 (5:5) in all groups.

Group 1: Pups were injected with saline (0.9%NaCl) (3 mL/kg, SC) parallel to VPA injection. Group 2: Pups were SC injected with VPA (300 mg/kg) to induce autism. Groups 3, 4, and 5: Pups were exposed to VPA (300 mg/kg, SC) with oral Cana at doses (5 mg/kg/day, 7.5 mg/kg/day, and 10 mg/kg/day) (Safhi et al., 2018; Abdelrahman et al., 2019) for PD-21 and continued during behavioral tests until PD-23 in volumes (6 mL/kg, 8 mL/kg, and 10 mL/kg). The behavioral experiments started at PD-21 to PD-23. Cana was administered by gastric gavages. Cana was administered 30 min before each behavioral test (Eissa et al., 2018).

The open field (OF) test was carried out on PD-21, followed by the elevated plus-maze test (EPM) on PD-22. Social behavior tests were performed on PD-23 (Mony et al., 2016; 2018). The pups were returned to the dams after completing all behavioral experiments (PD-23).

On PD-23, due to mortality, the number of each experimental group was 4 ± 1 for each sex except for the control group (no mortality). The ending number of pups was eight in all groups, except for the control group, which was 10. Rats were injected intraperitoneally with ketamine (80 mg/kg) (Salem et al., 2022) and sacrificed by cervical dislocation. Then brains were dissected out and washed with ice-cold saline. The cerebellum and cerebrum of each rat were isolated. Specimens from dissected brain tissues (cerebellum and cerebrum) were prepared for biochemical analysis (Eissa et al., 2018). One hemisphere of the cerebellum was fixed in neutral formalin for histopathological assessments (Samimi and Amin Edalatmanesh, 2016; Shona et al., 2018).

### 2.5 Behavioral assessment

#### 2.5.1 Three-chamber test (3C)

The test apparatus is a wooden box with three chambers (40 cm × 20 cm × 22 cm), and the sided chambers are separated from the center one with two openings for exploring the chambers. The test consisted of three sessions and was performed according to the process stated by DeVito et al. (2009) and Eissa et al. (2018). In the first session, a tested rat was habituated for 10 min during which it was placed in the central chamber and allowed to freely explore the empty apparatus. A sociability session of 5 min followed the habituation; a novel rat (the same age and with no previous contact with the tested rat) of the same strain was introduced inside the wire cage of one of sided chambers (novel rat zone). An identical empty wire cage was placed in the other sided chamber (novel object zone). Then the tested rat was placed in the central chamber and allowed to explore the sided chambers. The number of instances and time that the tested rat spent in direct contact with the novel rat (the time spent in grooming, running toward, sniffing or interacting, and crawling over the wired cage) in seconds (Sec), the time spent exploring the novel rat against the novel object (time spent in each chamber) in seconds, and the time spent close to against time spent far from the novel rat in seconds were measured. In the final social novelty session, another novel rat was introduced in the previous empty wire cage (novel rat zone), while the other chamber with the familiar rat was used in the previous sociability session (familiar rat zone). The same parameters were measured as with the previous session for 5 min (Lin et al., 2018; Lu et al., 2018). Behaviors were videotaped alternatively on two sets according to the time schedule to assess sociability and social preference. The measuring parameters were quantified by two observers to videotape blind to treatment conditions (Sailer et al., 2019).

#### 2.5.2 Elevated plus-maze (EPM) test

The maze test, which consisted of crossed two opened arms (30 cm × 10 cm) and two closed arms (30 cm × 10 cm × 15 cm) at 50 cm height from the floor, was performed following the methods stated by Pellow and File (1986) and Holmes et al. (2002). The rats were placed in the maze center to explore the maze for 10 min. The total number of entries and the time spent with the head and forepaws in seconds in either opened and closed arms of the maze (Eissa et al., 2018), grooming in addition to rearing frequency, and the numbers of both grooming and rearing/10 min (all time of the test) were measured. Behaviors were videotaped alternatively on two sets according to the time schedule to assess anxiety-like behaviors and exploratory behavior (Nguyen et al., 2017). The time spent and numbers of entries into each arm were quantified by two experimenters to videotape blind to treatment conditions (Sailer et al., 2019).

#### 2.5.3 Open field (OF) test

The apparatus was a black Plexiglas square box (60 cm × 60 cm × 30 cm height) with a black floor. The floor of the field was divided into 36 squares with a white marker (10 cm × 10 cm each). The test was conducted in a quiet place. Locomotion (the number of squares crossed by each rat as each entrance into a square of more than half the rat’s body) (Choi et al., 2018; Mansouri et al., 2019; Messiha et al., 2020) together with latency to leave the central area (in seconds) (Blázquez et al., 2019; Messiha et al., 2020), time spent in the central area (in seconds) and stereotype behaviors, grooming frequency and rearing (standing on the hind legs) frequency, and the numbers of both grooming and rearing/5 min (all time of the test) were measured. Behaviors were videotaped alternatively according to the time schedule on two sets to assess locomotor activity, exploratory behavior, anxiety-like behavior, and stereotype behaviors (Morakotsriwan et al., 2016; Messiha et al., 2020). Parameters were quantified by two experimenters blind to treatment conditions.

### 2.6 Light microscopic examination

One hemisphere of the cerebellum was fixed in 10% phosphate-buffered paraformaldehyde solution (pH = 7.4) for 18 h and then embedded in paraffin (Samimi and Amin Edalatmanesh, 2016; Shona et al., 2018). Tissues were sectioned at 5 μm thickness and left at 37°C to dry overnight. Then, sections were deparaffinized, rehydrated, and prepared for histopathological assessments. Cerebellar specimens were dehydrated in ascending grades of ethyl alcohol, cleared in xylol, embedded in paraffin wax, and sectioned at 5 μm thickness. Slides were stained with hematoxylin and eosin (H and E). Cerebellar specimens were examined, and the density of Purkinje cells in the cerebellum was scored (Shona et al., 2018). Sections were examined by a blinded investigator (Atef et al., 2019). The number of Purkinje cells was estimated in different parts of the cerebellar hemisphere (Shona et al., 2018).

### 2.7 Biochemical assessment

Other specimens from the dissected brain tissues were kept at −80°C and homogenized in ice-cold saline for biochemical analysis.

#### 2.7.1 Real-time quantitative polymerase chain reaction (RT-qPCR)

To measure the gene expression of PPAR-γ, LDHA, PDK, and c-Myc in the rat cerebellum and cerebrum tissues, RNA was extracted using an RNA extraction kit (Thermo Scientific, Fermentas, #K0731) according to the manufacturer’s instructions. Using a Nanodrop NA-1000 UV/vis spectrophotometer (Thermo Fisher Scientific Inc., Wilmington, DE, United States), RNA purity and concentration were measured and then stored at −80°C. Messenger RNA (mRNA) transcript levels of PPAR-γ, LDHA, PDK, and c-Myc were quantified by real-time PCR using StepOne Plus^™^ Real-Time PCR thermal cycler (Applied Biosystems, Waltham, MA, United States). RT-qPCR was performed using GoTaq^®^ 1-Step RT-qPCR System. Primers used are listed in Table 1. The thermal PCR amplification protocol was as follows: 37°C for 15 min, 10 min at 95°C, followed by 40 cycles of 95°C for 10 s, 52°C for 30 s, and 72°C for 30 s. The generation of specific PCR products was confirmed through dissociation curve analysis. Threshold (Ct) values for each reaction were estimated. All the Ct values of the target genes were normalized to the Ct value of β-actin, which was used as a housekeeping gene.

**TABLE 1:** | Primer sequences used in quantitative reverse transcription-polymerase chain reaction.

Gene	Forward sequence 5′-3′	Reverse sequence 5′-3′
PPAR-γ	GCC​AAG​AAC​ATC​CCC​AAC​TTC	GCA​AAG​ATG​GCC​TCA​TGC​A
LDHA	ATG​GCA​ACT​CTA​AAG​GAT​CAG​C	CCA​ACC​CCA​ACA​ACT​GTA​ATC​T
PDK	CGC​CAC​TCT​CCA​TGA​AGC​A	AAC​GAG​GTC​TTT​TCA​CAA​GCA​TT
c-Myc	CTG​CTG​TCC​TCC​GAG​TCC​TC	GGG​GGT​TGC​CTC​TTT​TCC​AC
β-actin	AAG​TCC​CTC​ACC​CTC​CCA​AAA​G	AAG​CAA​TGC​TGT​CAC​CTT​CCC

#### 2.7.2 Western blotting analysis

For GLUT-1 and PTEN detection, the cerebellum and cerebrum were homogenized in ice-cold RIPA lysis buffer containing protease and phosphatase inhibitors to preserve the protein integrity. Then, the lysates were centrifuged at 16,000 g for 10 min, and the supernatants were stored at −80°C. Prior to loading, protein levels were measured using the Bradford assay (Bradford, 1976). The lysate was mixed with an equal amount of 2 × Laemmli sample buffer and then boiled for 5 min to confirm protein denaturation, sonicated for half a min, and centrifuged at 10,000 g for 10 min. Next, the supernatants were loaded to 12% sodium dodecyl sulfate–polyacrylamide gel electrophoresis. Proteins were moved to PVDF membranes using a Bio-Rad Trans-Blot Turbo unit (Bio-Rad Laboratories Ltd., Watford, United Kingdom). The membrane was blocked for an hour in Tris-buffered saline (TBS) containing 5% (wt/vol) non-fat dry milk. Afterward, the membranes were incubated with primary antibodies against GLUT-1 (catalog # ab115730, Abcam) (Waltham, MA, United States) and PTEN (catalog # sc-377573, Santa Cruz Biotechnology) (Dallas, TX, United States) (1:1,000 dilution in TBS-T with 5% non-fat milk) at 4°C overnight. Blots were three times in TBS-T and incubated with the HRP-conjugated secondary antibody (goat anti-rabbit IgG-HRP-lmg goat mab, Novus Biological, 1:5,000 dilution). The signals were visualized with chemiluminescence according to the manufacturer’s protocol (Atef et al., 2019).

#### 2.7.3 ACh concentrations using ELISA kit

Cerebrum and cerebellum ACh concentrations were measured by the colorimetric method using sandwich ELISA Kits (catalog #E4452, BioVision Inc.^®^) (catalog # ab287811) (Milpitas, CA, United States), and expressed as µmol/mg protein according to the manufacturer’s instructions.

### 2.8 Statistical analysis

Statistical analyses were performed using GraphPad Prism 9.3.1., (471) (San Diego, CA, United States). Data of the current study were expressed as mean ± S.E.M. Quantitative variables were evaluated using one-way ANOVA followed by Tukey’s *post hoc* multiple comparisons test. Some behaviors tests, including time spent to explore novel object vs. novel rat, time spent close to novel rat vs. time spent far from novel rat, time spent to explore familiar rat vs. novel rat, time spent close to novel rat vs. time spent far from novel rat, time spent in opened arms vs. closed arms with the number of entries in opened arms vs. closed arms of EPM, were analyzed using two-way ANOVA, followed by Tukey’s *post hoc* multiple comparisons test after assessing the normality by the Shapiro–Wilk test or Kolmogorov–Smirov test.

## 3 Results

### 3.1 Effect of canagliflozin on mortality percentage

Pups injected with VPA (300 mg/kg, SC) twice daily on PD-2 and PD-3, and once daily on PD-4 resulted in an increase in the percentage of mortality (20%) compared to a mortality percentage equals 0% in vehicle-treated rats. Treatment with canagliflozin (5 mg/kg, 7.5 mg/kg, and 10 mg/kg) for 21 days—starting from the first day of VPA injection—did not significantly improve the mortality of rat pups compared to the VPA group (Table 2).

**TABLE 2:** Effect of canagliflozin (5 mg/kg, 7.5 mg/kg, and 10 mg/kg) on mortality percentage.

Groups	Mortality (percentage) %
Control	0
VPA	20^a^
VPA + Cana (5 mg/kg)	20
VPA + Cana (7.5 mg/kg)	20
VPA + Cana (10 mg/kg)	20

VPA, valproic acid; Cana, canagliflozin in VPA-induced autism in rats. Values are expressed as mean ± S.E.M and analyzed using one-way ANOVA, followed by Tukey’s *post hoc* multiple comparisons test, *n* = 8 for all groups except for the control group, *n* = 10.

^a^ Compared to the corresponding control group at *p* < 0.05.

### 3.2 Effect of canagliflozin on behavior

#### 3.2.1 Three-chamber test (3C)

##### 3.2.1.1 Effect of canagliflozin on sociability

**FIGURE 1:**
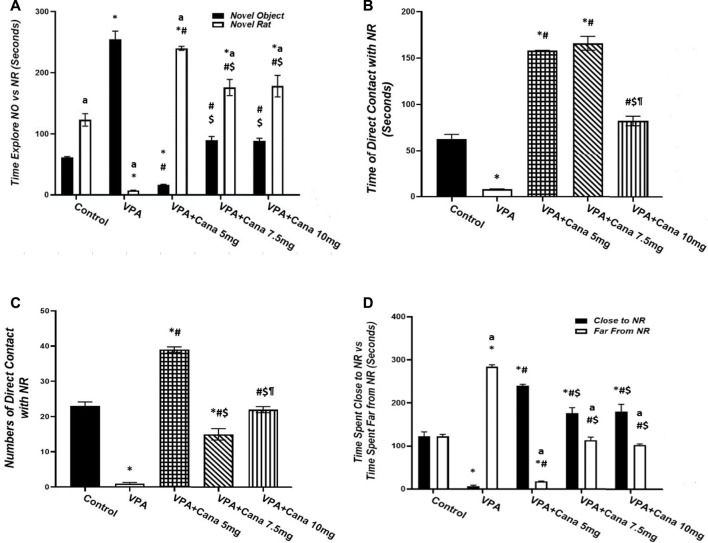
Effect of canagliflozin (5 mg/kg, 7.5 mg/kg, and 10 mg/kg) on sociability test; time explore NO *versus* NR **(A)**, time of direct contact with NR **(B)**, number of times of direct contact with NR **(C)**, and time spent close to NR *versus* time spent far from NR **(D)** in VPA-induced autism in rats. VPA, valproic acid; Cana, canagliflozin; NO, novel object; NR, novel rat. All values are expressed as mean ± S.E.M. Results represented in Figures 1B, C were analyzed using one-way ANOVA, followed by Tukey’s *post hoc* multiple comparisons test. Results represented in Figures 1A, D were analyzed using two-way ANOVA, followed by Tukey’s *post hoc* multiple comparisons test. ^*^Compared to the corresponding control group at *p* < 0.05, ^#^ compared to the corresponding VPA group at *p* < 0.05, ^$^ compared to the corresponding VPA + Cana (5 mg/kg) group at *p* < 0.05, ^¶^ compared to the corresponding VPA + Cana (7.5 mg/kg) group, ^a^ compared to time exploring NO within the same experimental groups **(A)**, ^a^ compared to time close to novel rat **(D)** at *p* < 0.05, *n* = 8, for all groups except for the control group, *n* = 10.

All groups other than the VPA-treated group presented more preference toward the novel rat than the object (empty cage) (Figure 1A). Pups injected with VPA (300 mg/kg, SC) on PD-2 and PD-3 twice daily, and on PD-4 once daily, spent more time exploring the novel object and less time exploring the novel rat than vehicle-injected rats (*p* < 0.05, Figure 1A). Nevertheless, oral administration of Cana (5 mg/kg, 7.5 mg/kg, and 10 mg/kg) reduced the time spent exploring the novel object, while it increased the time spent exploring the novel rat in comparison to the induction group (*p* < 0.05, Figure 1A). A Cana dose of 5 mg/kg showed the greatest effect compared with the VPA-treated group (*p* < 0.05, Figure 1A).

Injection of VPA (300 mg/kg, SC) resulted in less time spent and decreased the frequency of direct contact with the novel rat compared with the vehicle-treated group (*p* < 0.05, Figures 1B, C). Furthermore, Cana (5 mg/kg, 7.5 mg/kg, and 10 mg/kg) increased the time spent in direct contact with a novel rat when compared with the VPA-injected group (*p* < 0.05, Figures 1B, C). Cana (5 mg and 7.5 mg)-treated groups directed the greatest effect of time spent in direct contact with the novel rat (Figure 1B), while Cana 5 mg pointed to the greatest number of direct contacts with the novel rat compared to the VPA-treated group (*p* < 0.05, Figure 1C).

Valproic acid injected SC at a dosage of 300 mg/kg decreased time spent close to, and increased time spent far from, the novel rat compared to the vehicle-treated group (*p* < 0.05, Figure 1D). However, coadministration of oral Cana at doses of 5 mg/kg, 7.5 mg/kg, and 10 mg/kg with VPA increased the time spent in close contact with, and decreased the time spent far from, a novel rat compared with the VPA-treated group (*p* < 0.05, Figure 1D). A Cana dose of 5 mg presented the longest time spent close to the novel rat and the shortest time spent far from the novel rat compared to the VPA-treated group (*p* < 0.05, Figure 1D).

##### 3.2.1.2 Effect of canagliflozin on social novelty

**FIGURE 2:**
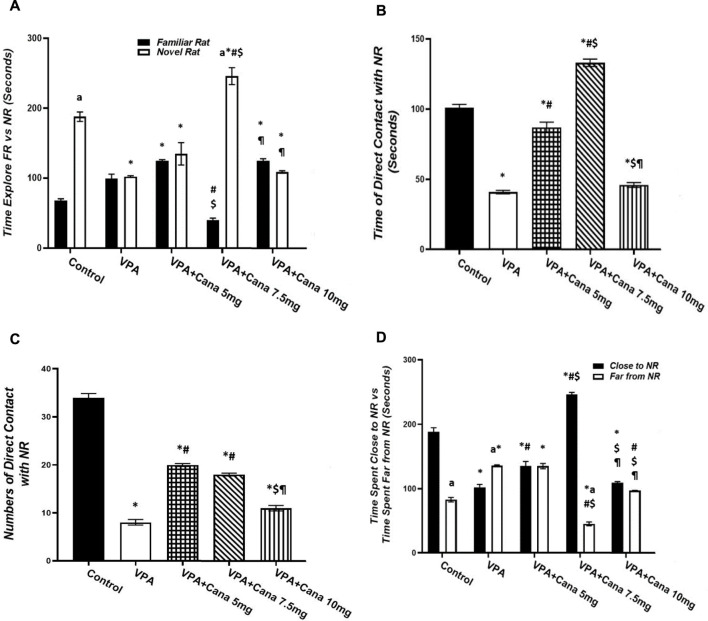
Effect of canagliflozin (5 mg/kg, 7.5 mg/kg, and 10 mg/kg) on social novelty test, time explore FR *versus* NR **(A)**, time of direct contacts with NR **(B)**, number of times of direct contact with NR **(C)**, and time spent close to NR *versus* time spent far from NR **(D)** in VPA-induced autism in rats. VPA, valproic acid; Cana, canagliflozin; FR, familiar rat; NR, novel rat. Values are expressed as mean ± S.E.M. Results represented in Figures 2B, C were analyzed using one-way ANOVA, followed by Tukey’s *post hoc* multiple comparisons test. Results represented in Figure 2A, D were analyzed using two-way ANOVA, followed by Tukey’s *post hoc* multiple comparisons test. *Compared to the corresponding control group at *p* < 0.05, ^#^ compared to the corresponding VPA group at *p* < 0.05, ^$^ compared to the corresponding VPA + Cana (5 mg/kg) group at *p* < 0.05, ^¶^ compared to the corresponding VPA + Cana (7.5 mg/kg) group, ^a^ compared to time exploring FR within the same experimental groups **(A)**, ^a^ compared to Time Close to NR **(D)** at *p* < 0.05, *n* = 8, for all groups except for the control group, *n* = 10.

Pups injected with VPA (300 mg/kg, SC) twice daily on PD-2 and PD-3, and once daily on PD-4, spent even time exploring both familiar and novel rats, and, conversely, spent less time exploring novel rats in comparison to the vehicle-treated group (*p* < 0.05, Figure 2A). Nevertheless, oral administration of only Cana (7.5 mg/kg) increased the time spent exploring novel rats and decreased the time spent exploring familiar rats compared to the VPA-treated group (*p* < 0.05, Figure 2A).

Valproic acid injected SC at a dosage of 300 mg/kg resulted in less time spent and a reduced number of direct contact with novel rats compared with vehicle-injected rats (*p* < 0.05, Figures 2B, C). However, coadministration of Cana at doses of 5 and 7.5 mg/kg with VPA increased the time spent in direct contact with novel rats compared to the VPA-treated group (*p* < 0.05, Figures 2B, C).

Injection of VPA (300 mg/kg, SC) reduced the time spent close to novel rats compared to the vehicle-treated group (*p* < 0.05, Figure 2D). Furthermore, Cana (5 mg/kg and 7.5 mg/kg) amplified the time spent close to novel rats compared with the VPA-treated group (*p* < 0.05, Figure 2D). In the opposite way, the VPA-injected group augmented the time spent far from novel rats compared with the vehicle-injected group (*p* < 0.05, Figure 2D). Coadministration of Cana paralleled with VPA injection at doses of 7.5 mg/kg and 10 mg/kg decreased the time spent far from novel rats compared to the VPA-injected group (*p* < 0.05, Figure 2D). Oral administration of a Cana dose of 7.5 mg exhibited the greatest increase in the time spent close to novel rats and the greatest decrease in the time spent far from novel rats compared with the VPA-treated group (*p* < 0.05, Figure 2D).

#### 3.2.2 Elevated plus-maze test (EPM)

**FIGURE 3:**
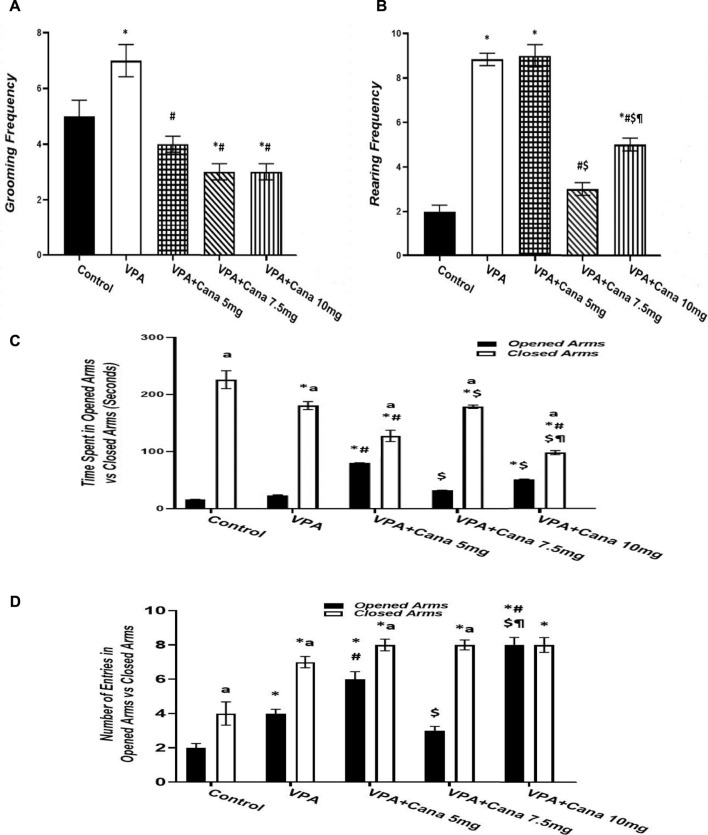
Effect of canagliflozin (5 mg/kg, 7.5 mg/kg, and 10 mg/kg) on elevated plus-maze (EPM) test; grooming **(A)**, rearing **(B)**, time spent in opened arms *versus* closed arms **(C)**, and number of entries in opened arms *versus* closed arms **(D)**. VPA, valproic acid; Cana, canagliflozin. Values are expressed as mean ± S.E.M. Results represented in Figures 3A, B were analyzed using one-way ANOVA, followed by Tukey’s *post hoc* multiple comparisons test. Results represented in Figures 3C, D were analyzed using two-way ANOVA, followed by Tukey’s *post hoc* multiple comparisons test. ^*^ Compared to the corresponding control group at *p* < 0.05, ^#^ compared to the corresponding VPA group at *p* < 0.05, ^$^ compared to the corresponding VPA + Cana (5 mg/kg) group at *p* < 0.05, ^¶^ compared to the corresponding VPA + Cana (7.5 mg/kg) group, ^a^ compared to time spent in opened arms **(C)** and number of entries in opened arms **(D)** at *p* < 0.05, *n* = 8, for all groups except for the control group, *n* = 10.

Pups injected with VPA (300 mg/kg) raised the number of groomings and rearings compared to the vehicle-injected group (*p* < 0.05, Figures 3A, B), whereas oral administration of Cana (5, 7.5, and 10 mg/kg) inhibited the number of groomings compared with the VPA-treated group (*p* < 0.05, Figure 3A). In addition, Cana at doses of 7.5 mg and 10 mg revealed the same lowest number of groomings (*p* < 0.05, Figure 3A), while only Cana doses of 7.5 m/kg and 10 m/kg reduced the number of rearings in comparison with the VPA-treated group (*p* < 0.05, Figure 3B), with a dose of 7.5 mg/kg revealing the lowest number of rearings.

Valproic acid injection at a dose of 300 mg/kg inhibited the time spent in closed arms with no significant change in the time spent in opened arms compared to the vehicle-treated group (*p* < 0.05, Figure 3C). A Cana dose of 5 mg/kg amplified the time spent in opened arms in comparison to the VPA-treated group (*p* < 0.05, Figure 3C). Furthermore, Cana (5 mg/kg and 10 mg/kg) reduced the time spent in closed arms compared with the VPA-treated group (*p* < 0.05, Figure 3C). All groups intensified the latency in closed arms *versus* in opened arms (Figure 3C).

SC injection of VPA twice daily on PD-2 and PD-3 and once daily on PD-4 raised the number of entries in both opened arms and closed arms compared to the vehicle-treated group (*p* < 0.05, Figure 3D). Treatment with oral Cana (5 mg/kg and 10 mg/kg) augmented the number of entries in opened arms without a change in the number of entries within closed arms compared with the VPA-injected group (*p* < 0.05, Figure 3D). All groups except for the VPA + Cana 10 mg/kg group increased the number of entries in closed arms compared to opened arms.

#### 3.2.3 Open field test (OF)

**FIGURE 4:**
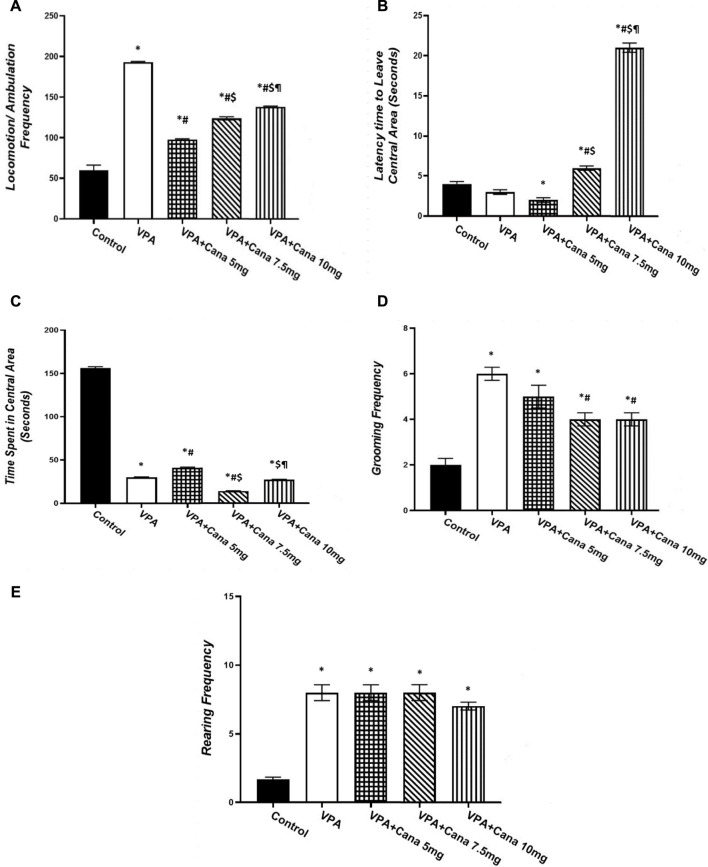
Effect of canagliflozin (5 mg/kg, 7.5 mg/kg, and 10 mg/kg) on open field (OF) test; locomotion **(A)**, latency to leave central area **(B)**, time spent in central area **(C)**, grooming **(D)**, and rearing **(E)** in VPA-induced autism in rats. VPA, valproic acid; Cana, canagliflozin. Values are expressed as mean ± S.E.M and analyzed using one-way ANOVA, followed by Tukey’s *post hoc* multiple comparisons test. ^*^ Compared to the corresponding control group at *p* < 0.05, ^#^ compared to the corresponding VPA group at *p* < 0.05, ^$^ compared to the corresponding VPA + Cana (5 mg/kg) group at *p* < 0.05, ^¶^ compared to the corresponding VPA + Cana (7.5 mg/kg) group at *p* < 0.05, *n* = 8, for all groups except for the control group, *n* = 10.

Valproic acid injection to pups at a dose of 300 mg/kg raised locomotor activity (*p* < 0.05, Figure 4A), inhibited the time spent in the central area (*p* < 0.05, Figure 4C), and increased the number of groomings and rearings compared to the vehicle-treated rats (*p* < 0.05, Figures 4D, E). Oral treatment with Cana (5 mg/kg, 7.5 mg/kg, and 10 mg/kg) reduced locomotion compared with the VPA-injected group (*p* < 0.05, Figure 4A), with Cana (5 mg) displaying the highest decrease. Moreover, Cana (7.5 mg/kg and 10 mg/kg) prolonged the time to leave the central area in comparison with the VPA-treated group (*p* < 0.05, Figure 4B), with Cana (10 mg) pointing to the highest increase. In addition, Cana (7.5 mg and 10 mg) showed the same significant decrease in groomings compared with the VPA-treated group (*p* < 0.05, Figure 4D). However, Cana at a 5 mg/kg dose only increased the time spent in the central area compared to the VPA-treated group (*p* < 0.05, Figure 4C). All Cana doses displayed no significant difference in rearings compared to the induction group (Figure 4E).

### 3.3 Effect of canagliflozin on VPA-induced histopathological changes

**FIGURE 5:**
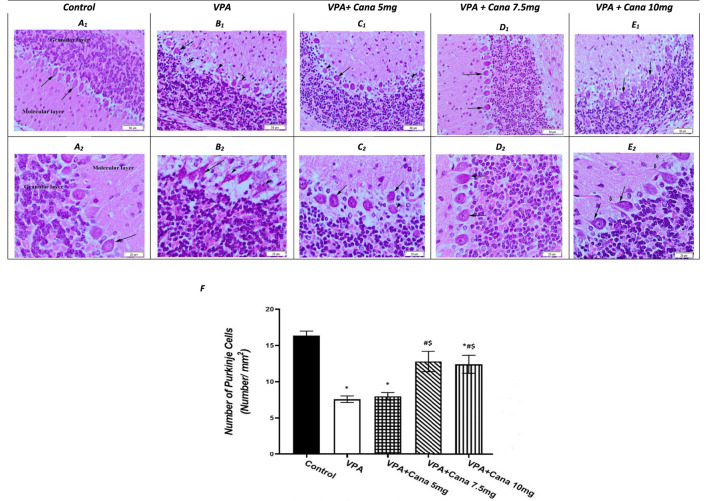
Effect of canagliflozin (5 mg/kg, 7.5 mg/kg, and 10 mg/kg) on the histopathological picture of the cerebellum tissues of the brain in VPA-induced autism in rats. Hematoxylin and eosin stain (50 µm and 20 µm) **(A–E)**. Number of Purkinje cells of the experimental groups **(F)**. VPA, valproic acid; Cana, canagliflozin. Values are expressed as mean ± S.E.M and analyzed using one-way ANOVA, followed by Tukey’s *post hoc* multiple comparisons test. ^*^ Compared to the corresponding control group at *p* < 0.05, ^#^ compared to the corresponding VPA group at *p* < 0.05, ^$^ compared to the corresponding VPA + Cana (5 mg/kg) group at *p* < 0.05, ^¶^ compared to the corresponding VPA + Cana (7.5 mg/kg) group at *p* < 0.05, *n* = 8, for all groups except for the control group, *n* = 10.

Histopathological evaluation of control group (group I) specimens, with H and E stain, presented the normal cerebellar cortex layers, and the molecular and the Purkinje cell with the granular layers (Figure 5A). The molecular layer appeared as a pale zone with few stellate cells. The Purkinje layer contained a large number of Purkinje cells with a single row of intact oval or flask-shaped cell bodies and a large, rounded vesicular normal central nucleus with a regular intact envelope (Figures 5A, F) as well as a huge number of myelinated axons (Figure 5A). The internal granular layer displayed small, deeply stained granular cells, while the external granular layer presented with small, closely packed cells with a deeply stained, normally rounded nucleus (Figure 5A).

Valproic acid injections to pups at a dose of 300 mg/kg displayed a neurotoxic effect on the cerebellum Purkinje cells. Purkinje cells displayed lowered density, and marked depletion and degeneration. In addition, they appeared shrunken and disorganized in the uneven cell membrane which enclosed vacuolated spaces (empty haloes) (Figures 5B, F). Nuclear damage of the nuclei looked shrunken with an irregular nuclear envelope (Figure 5B). Degenerated axons were located completely vacuolated with a reduction of the myelin sheath (Figure 5B). A large number of degenerated Purkinje cells, along with degenerated swollen vacuolated axons, lacked neurofilaments and organelles (Figure 5B).

The molecular and internal granular layers exhibited massive reduction (Figure 5B). The molecular layers enclosed deeply stained, scattered basket cells (Figure 5B). The internal granular layer presented with small cells packed within congested intercellular spaces (Figure 5B). An external granular layer was noticed on the cerebellar surface in some sections that showed degenerative changes in the cerebellar cortex of deeply stained cells (Figure 5B).

The experimental groups co-treated with VPA and Cana (III, IV, and V) established recovery of the degenerated cerebellar construction that appeared in the VPA-treated group (Figures 5C–E). Cana treatment preserved the normal arrangement construction of the Purkinje cells layer with intact myelinated axons and nuclei (Figures 5C–E). Purkinje cells returned to their normal oval flask-shaped appearance and number, and were arranged in a single row (Figures 5C–E). The layer’s thickness and normal arrangement of the cerebellar cortex mimicked a histopathological picture of the control group (Figures 5C–E). The molecular layer demonstrated scattered basket cells. The internal and external granular layers appeared normal with a totally intact nucleus and cytoplasm (Figures 5C–E).

### 3.4 Effect of canagliflozin on PPAR-γ, LDHA, PDK, and c-Myc gene expression

**FIGURE 6:**
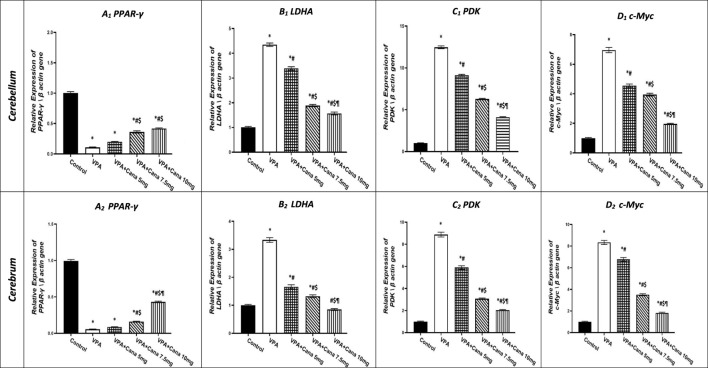
Effect of canagliflozin (5 mg/kg, 7.5 mg/kg, and 10 mg/kg) on the expression of PPAR-γ **(A)**, LDHA **(B)**, PDK **(C)**, and c-Myc **(D)** gene in the cerebellum and cerebrum tissues of VPA-induced autism in rats. VPA, valproic acid; Cana, canagliflozin. Results are expressed as mean ± S.E.M and analyzed using one-way ANOVA, followed by Tukey’s *post hoc* multiple comparisons test. ^*^ Compared to the corresponding control group at *p* < 0.05, ^#^ compared to the corresponding VPA group at *p* < 0.05, ^$^ compared to the corresponding VPA + Cana (5 mg/kg) group at *p* < 0.05, ^¶^ compared to the corresponding VPA + Cana (7.5 mg/kg) group at *p* < 0.05, *n* = 8, for all groups except for the control group, *n* = 10.

Injection with VPA of 300 mg/kg developed a downregulation in the gene expression of PPAR-γ in the cerebellum by 90% and cerebrum tissues of the brain by 95% compared to the vehicle-treated group (*p* < 0.05, Figure 6A). Oral administration of Cana doses of 7.5 mg/kg and 10 mg/kg upregulated gene expression of PPAR-γ compared to the VPA-treated group in both the cerebellum by (2, 3, and 4 folds, respectively) and cerebrum tissues of the brain by 1.5, 3, and 9 folds, respectively (*p* < 0.05, Figure 6A). Furthermore, treatment with Cana at a dose of 10 mg/kg displayed the highest upregulation in gene expression to reach almost 9 folds greater than the VPA group in cerebrum tissues (*p* < 0.05, Figure 6A_2_), while both Cana groups (7.5 mg and 10 mg) showed almost the same upregulation level of PPAR-γ in the cerebellum compared with the VPA-treated group (*p* < 0.05, Figure 6A_1_).

On the other hand, SC injection with VPA resulted in the upregulation of LDHA gene expression in both the cerebellum and cerebrum tissues of the brain compared to the vehicle-treated group (*p* < 0.05, Figure 6B). The expression of LDHA increased by 4 folds in the cerebellum and 3 folds in the cerebrum tissues isolated from VPA-treated animals. Oral administration of Cana (5 mg/kg, 7.5 mg/kg, and 10 mg/kg) downregulated the gene expression of LDHA compared to the VPA group in both the cerebellum by 22%, 64%, and 73%, respectively, and cerebrum tissues of the brain by 51%, 60%, and 75%, respectively (*p* < 0.05, Figure 6B). Furthermore, Cana (10 mg/kg) established the highest downregulation of LDHA gene expression among all treatment groups compared to the induction group in the cerebellum and cerebrum (*p* < 0.05, Figure 6B).

Injection with VPA twice a day on PD2 and PD-3, and once daily on PD-4, with a dose of 300 mg/kg caused upregulation in the gene expression of PDK by 12 folds in the cerebellum and cerebrum tissues of the brain by 9 folds compared with the vehicle group (*p* < 0.05, Figure 6C). Administration of Cana (5 mg/kg, 7.5 mg/kg, and 10 mg/kg) downregulated the gene expression of PDK compared to the VPA group in both the cerebellum by 27%, 50%, and 67%, respectively, and cerebrum tissues of the brain by 34%, 66%, and 77%, respectively (*p* < 0.05, Figure 6B). Moreover, Cana (10 mg/kg) presented the highest downregulation in PDK gene expression by 75% among all treatment groups compared to the VPA-injected group in the cerebellum and cerebrum (*p* < 0.05, Figure 6B).

Injection with VPA (300 mg/kg, SC) resulted in a higher upregulation of c-Myc gene expression in the cerebellum by 7 folds and cerebrum tissues of the brain by 8 folds compared with the vehicle-treated group (*p* < 0.05, Figure 6D). Cana treatment doses of 5 mg/kg, 7.5 mg/kg, and 10 mg/kg downregulated c-Myc gene expression compared with the VPA-treated group in both the cerebellum by 35, 44%, and 72%, respectively, and cerebrum tissues of the brain by 19%, 58%, and 79%, respectively (*p* < 0.05, Figure 6D). Furthermore, treatment with Cana (10 mg/kg) presented the highest downregulation in c-Myc gene expression within all treatment groups compared with the VPA-treated group in both the cerebellum (79%) and cerebrum (72%) (*p* < 0.05, Figure 6D).

### 3.5 Effect of canagliflozin on GLUT-1 and PTEN protein expression

**FIGURE 7:**
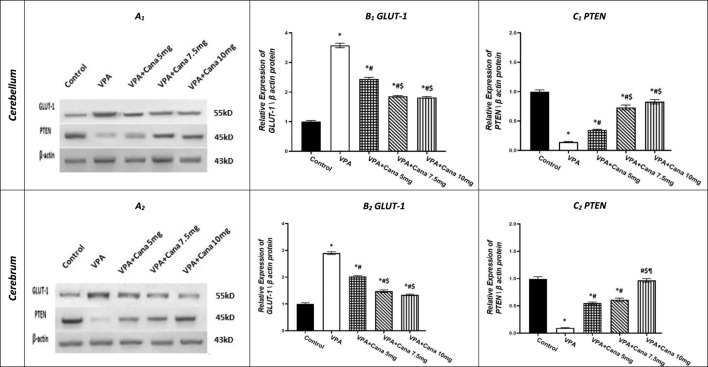
Effect of canagliflozin (5 mg/kg, 7.5 mg/kg, and 10 mg/kg) on the expression of GLUT-1 **(B)** and PTEN **(C)** protein in the cerebellum and cerebrum tissues of VPA-induced autism in rats. VPA, valproic acid; Cana, canagliflozin. Results are expressed as mean ± S.E.M and analyzed using one-way ANOVA, followed by Tukey’s *post hoc* multiple comparisons test. ^*^ Compared to the corresponding control group at *p* < 0.05, ^#^ compared to the corresponding VPA group at *p* < 0.05, ^$^ compared to the corresponding VPA + Cana (5 mg/kg) group at *p* < 0.05, ^¶^ compared to the corresponding VPA + Cana (7.5 mg/kg) group at *p* < 0.05, *n* = 8, for all groups except for the control group, *n* = 10.

Injection with SC VPA twice daily on PD-2 and PD-3, and once on PD-4, resulted in increase in the protein expression of GLUT-1 in the cerebellum by 4 folds and cerebrum tissues of the brain by 3 folds compared to the vehicle-treated group (*p* < 0.05, Figures 7A, B). However, Cana administration with doses of 5 mg/kg, 7.5 mg/kg, and 10 mg/kg reduced the protein expression of GLUT-1 compared to the VPA group in both the cerebellum by 32%, 48%, and 49%, respectively, and cerebrum tissues by 30%, 50%, and 54%, respectively, of the brain (*p* < 0.05, Figures 7A, B). Furthermore, oral Cana at doses of 7.5 mg/kg and 10 mg/kg displayed the same highest decline in protein expression to reach almost 50% compared to the VPA group (*p* < 0.05, Figures 7A, B) in the cerebellum and cerebrum tissues of the brain.

Conversely, VPA injected into pups at a dosage of 300 mg/kg SC reduced the protein expression of PTEN in the cerebellum by 86% and cerebrum tissues of the brain by 91% compared to the vehicle-treated group (*p* < 0.05, Figures 7A, C). Protein expression of PTEN increased in Cana groups (5 mg/kg, 7.5 mg/kg, and 10 mg/kg) compared to the VPA group in both the cerebellum by 2, 5, and 6 folds, respectively, and cerebrum tissues of the brain by 6, 6, and 10, respectively (*p* < 0.05, Figures 7A, C). Furthermore, Cana group V (10 mg/kg) presented the highest increase in protein expression compared to the VPA-induced group in the cerebrum (10 folds) (*p* < 0.05, Figure 7.A_2_, C_2_), while both Cana doses of 7.5 and 10 mg showed the same increase of protein expression in the cerebellum (5 and 6 folds) compared to the VPA-treated group (*p* < 0.05, Figures 7A_1_, C_1_).

### 3.6 Effect of canagliflozin on tissue ACh levels

**FIGURE 8:**
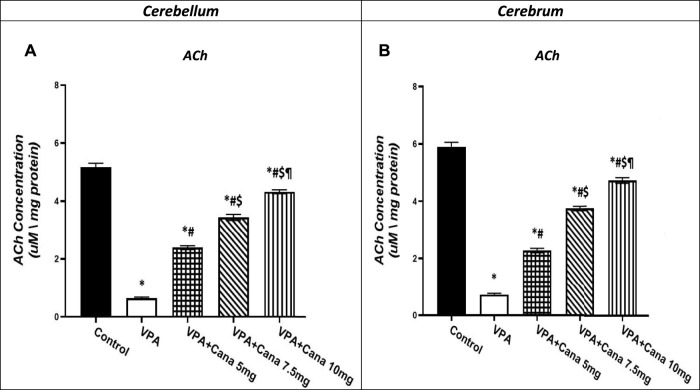
Effect of canagliflozin (5, 7.5, and 10 mg/kg) on cerebellum **(A)** and cerebrum **(B)** tissue level of ACh in VPA-induced autism in rats. VPA: valproic acid, Cana: canagliflozin. Results are expressed as mean ± S.E.M and analyzed using one-way ANOVA followed by Tukey post hoc multiple comparisons test. ^*^ Compared to the corresponding Control group at *P* < 0.05, ^#^ Compared to the corresponding VPA group at *P* < 0.05, ^$^ Compared to the corresponding VPA + Cana (5 mg/kg) group at *P* < 0.05, ^¶^ Compared to the corresponding VPA + Cana (7.5 mg/kg) group at *P* < 0.05, *n* = 8, for all groups except for control group, *n* = 10.

Valproic acid-injected rats (300 mg/kg, SC) addressed the reduction of ACh levels by 88% in the cerebellum and the cerebrum tissues compared to vehicle-injected rats (*p* < 0.05, Figures 8A, B). Administration of Cana at doses of 5 mg/kg, 7.5 mg/kg, and 10 mg/kg increased ACh levels by 4, 5, and 7 folds, respectively, in the cerebellum and cerebrum tissues compared to the VPA-treated group (*p* < 0.05, Figures 8A, B). Cana 10 mg showed the highest surge in ACh levels among all treatment groups to reach 7 folds the concentration of ACh in the VPA-treated group (*p* < 0.05, Figures 8A, B).

## 4 Discussion

In this behavioral study, subcutaneous injection of VPA resulted in several changes in behavioral and biochemical analyses that mimicked the changes that occur in autistic patients (Favre et al., 2013; Larner et al., 2021). Rat pups selected in the present study were of both sexes based on the study by Rasalam et al. (2005), which confirmed that there were no differences in the prevalence of autism in VPA-exposed children during pregnancy and considered a 1:1 male-to-female ratio (Rasalam et al., 2005).

In agreement with previous studies, numerous structural and functional changes either in the cerebral cortex or cerebellum were detected in VPA-induced ASD in rats and mice models (Schneider and Przewłocki, 2005; Wagner et al., 2006), with some resemblance to the changes studied in autistic children’ brains. Valproic acid significantly reduced the numbers and sizes of Purkinje cells in the cerebellum (Morakotsriwan et al., 2016). Similarly, prenatal VPA exposure induced remarkable cerebellar Purkinje and granular layers with axon degeneration (Hafez et al., 2018; Shona et al., 2018). Furthermore, Purkinje cell atrophy confirmed the neurotoxic effect of repeated VPA exposure on the cerebellar structure and function (Main and Kulesza, 2017).

Early postnatal cerebellum lesions increased spontaneous motor activity in rats (Bobée et al., 2000). Furthermore, the loss of Purkinje cells in mice induced significantly increased repetitive behaviors (Martin et al., 2010). Studies in rodents confirmed a vital role of the cerebellum in motor, repetitive, and exploratory behavioral deficits or anxiety-like behaviors as observed in autism (Pierce and Courchesne, 2001). The cerebellum defect resulted in many psychotic disorders, including anxiety (Baldaçara et al., 2008). Early prefrontal cortex (PFC) damage in humans impairs social interaction (Eslinger et al., 2004). Neonatal PFC lesions decreased social play and conditioned place preference associated with social contacts and social grooming in rats (Schneider and Koch, 2005).

VPA-treated rats displayed significantly decreased social behaviors, including sociability and social novelty indices (Mony et al., 2016; Morakotsriwan et al., 2016; Eissa et al., 2018). Valproic acid exposure impaired social abilities in a three-chamber social assay test in a VPA-induced rat model of autism (Chau et al., 2017; Wu et al., 2017; Rajizadeh et al., 2021). Similarly, the VPA-injected mice displayed fewer sociability and social preference behaviors (Roullet et al., 2010; Kim et al., 2014).

Rats injected with VPA showed no preference toward novel rats, either by spending more time with familiar rats and less time sniffing the novel rats compared with the saline-injected group (Campolongo et al., 2018; Larner et al., 2021) or by spending even time exploring novel and familiar mice, compared to the control group (Larner et al., 2021), and that is consistent with our findings.

Both sociability and social novelty behaviors’ studies confirmed the sociability deficits following early postnatal VPA. Furthermore, sociability and social memory are independent social behaviors which respond differently to environmental changes without any effect on each other, which is in line with a study that indicated autistic children tend to avoid social interaction (Kanner, 1943). Furthermore, the behaviors observed in children with ASD confirmed that autistic children are more secure and social with familiar individuals and objects (Mychasiuk et al., 2012). Impaired sociability is provoked *via* heightened anxiety or fear, which in turn leads to environmental fear stimuli (Stein et al., 2002; Tillfors, 2004).

Valproic acid-induced anxiety behavior is an autistic non-core symptom compared to control animals (Mohammadi et al., 2020). Moreover, VPA exposure from PD-2 to PD-4 stimulated both anxiety and hyperactivity behaviors in adolescent rats (Al-Amin et al., 2015; Mony et al., 2016). Early postnatal VPA exposure decreased the time spent in the center area while increasing locomotor activity compared with control mice (Bath and Pimentel, 2017). Similarly, the VPA-injected rats spent less time in the center of the open field, which is an indication of anxiety (Mohammadi et al., 2020; Larner et al., 2021). The pathological study demonstrated high rates of anxiety in VPA-exposed pediatrics (Glauser, 2004). Valproic acid exposure increased anxiety-like behaviors in EPM, which is demonstrated by spending more time in the closed arms in rats (Larner et al., 2021; Rajizadeh et al., 2021). Furthermore, disturbed anxiety levels and hyperactivity were observed in the VPA-treated group of mice in EPM (Eissa et al., 2018).

Valproic acid increases overall motor activity in rodents (Kim et al., 2014; Larner et al., 2021). Valproic acid induced an increase in the number of crossing bars of the OF test as a reflection of hyperlocomotion in rats (Schneider et al., 2008; Mony et al., 2016). The features of hyperactivity were reported in various mouse models of autism (Peñagarikano et al., 2011; Schmeisser et al., 2012).

Valproic acid treatment increased repetitive, stereotyped behavior measured in OF in rats as an ASD core symptom (Schneider and Przewłocki, 2005; Schneider et al., 2008). Prenatal VPA treatment significantly augmented the grooming and rearing number, and duration compared with the control group (Dai et al., 2018; Mohammadi et al., 2020). Mice injected with VPA increased repetitive-stereotyped movements with more time engaged compared to the control group (Zhang et al., 2012). Similarly, VPA-injected rats induced more grooming behaviors than those in the vehicle-treated group (Gandal et al., 2010; Mehta et al., 2011).

VPA resulted in social deficits in exposed mice, as changes in the ACh level triggered abnormal social, hyperactive, repetitive, and anxiety-like behaviors (Kim et al., 2014). Upregulation of acetylcholinesterase (AChE) protein expression was detected in both human and animal studies (Friedman et al., 2006; Karvat and Kimchi, 2014). Similarly, AChE expression increased in cultures treated with VPA (Kim et al., 2014). Furthermore, decreased levels of ACh in the prefrontal cortex resulted in attention deficit and impulsive behavior in mice (McTighe et al., 2013). ACh downregulation, a neurotransmitter for neuronal development in the brain (Picciotto et al., 2012), was apparently observed in the brain of ASD patients, which further resulted in behavioral changes in autistic patients (Petersen et al., 2013).

VPA administration altered PTEN expression in the brain (Ha et al., 2017). Similarly, prenatal VPA administration to mice reduced PTEN in the hippocampus and cortex, resulting in developmental delay and neuroanatomical changes (Yang et al., 2016). PTEN is downregulated in autistic glial cells (Zhou and Parada, 2012).

A deficiency of PTEN expression in the Purkinje cells of the cerebellum caused repetitive behavior, sociability deficits, and motor-learning defects in mice. PTEN-deficient mice displayed hyperactivity with impaired social activity (Ogawa et al., 2007). PTEN ± mice, detected in Purkinje cells, impaired sociability behaviors with deficits in motor learning (Lugo et al., 2014; Kwan et al., 2016). The downstream pathway of PTEN resulted in behavioral abnormalities and played a significant role in ASD (Clipperton-Allen and Page, 2014; Lugo et al., 2014).

Nuclear β-catenin translocation interacted with TCF/LEF, which stimulated the target genes, PDK and cMyc (Lecarpentier et al., 2017). Similarly, Wnt/β-catenin pathway over-activation stimulated aerobic glycolysis *via* induction of PDK (Lecarpentier et al., 2017; Vallée et al., 2017; Vallée and Vallée, 2018). PDK1, a glycolysis regulator, phosphorylated the PDH complex, inhibiting cetyl-CoA formation from pyruvate in mitochondria (Lecarpentier et al., 2017). Then, cytosolic pyruvate is directed for lactate formation and then released by LDHA and MCT-1 from the cell (Zhang et al., 2014).

c-Myc also activated LDHA, which stimulated the pyruvate conversion to lactate (Dang, 2010). Furthermore, the study indicated a significant increase in LDHA expression (Khemakhem et al., 2017) in ASD patients. PKM2 bound β-catenin *via* c-Myc in the nucleus for further induction of glycolytic enzyme expression of GLUT, LDHA, and PDK1 (Yang et al., 2012).

Downregulation of the Wnt/β-catenin pathway stimulated PPAR γ, while PPAR γ induction reduced the expression of β-catenin (Moldes et al., 2003; Jansson et al., 2005). In fact, both the Wnt/β-catenin pathway and PPAR γ counteract each other in various diseases, such as cancers (Vallée et al., 2017).

Co-administration of Cana with VPA improved the impaired behavior of VPA-treated rats, which could partly be explained by amplified ACh levels. The previous study confirmed that donepezil, the AChE inhibitor, rescued the autistic behaviors in VPA-treated mice *via* upregulation of the ACh level (Kim et al., 2014). Treatment with donepezil reduced impaired sociability, hyperactivity, anxiety-like behaviors, and repetitive digging behavior in mice treated with VPA (Kim et al., 2014). In addition, pre-treating mice with donepezil relieved anxiety by inhibiting the hyperactivity observed in EPM *via* attenuating the spending time as well as the entry number in opened arms, as an indication of having protective effects on cognitive functions in the VPA model of autism (Eissa et al., 2018). The changes in ACh levels in the cerebral cortex contributed to abnormal social and repetitive behaviors (Kim et al., 2014). The sociability index in the three-chamber test increased with donepezil administration through the elevation of ACh levels in mice (Karvat and Kimchi, 2014; Kim et al., 2014).

PPAR γ agonist stimulated PTEN expression (Vallée et al., 2017; Vallée and Vallée, 2018). Similarly, pioglitazone, as a PPAR γ agonist, recovered most of the typical behaviors of autism by correcting social as well as communication deficits in lipopolysaccharide (LPS)-induced autistic-like behaviors in rats (Kirsten et al., 2018; 2019). Pioglitazone improved behavior changes during adulthood in rats of the endotoxin model of autism (Kirsten et al., 2018). Furthermore, daily pioglitazone treatment effectively attenuates hyperactivity, stereotypic behaviors, irritability, and lethargy measured in autistic children without significant side effects (Boris et al., 2007).

Cana reduced the translocation of β-catenin in the nucleus (Hung et al., 2019). Similarly, PPAR γ agonists inhibited β-catenin; otherwise, PPAR γ was activated *via* canonical Wnt/β-catenin pathway inhibition (Lecarpentier et al., 2017). Furthermore, troglitazone, a PPAR γ agonist, reduced the level of c-Myc (Akinyeke and Stewart, 2011). Along the same line, PPAR γ activation selectively decreased PDK mRNA (Abbot et al., 2005). Clinical trials that studied pioglitazone suggested that PPARs be targeted for drug therapy of ASD (Boris et al., 2007; Ghaleiha et al., 2015).

Pioglitazone improved glucose utilization in addition to lactate production in brain glial cells (Pilipović et al., 2015). When increasing the dose, Cana acted on SGLT2 in addition to other glucose transporters, mainly GLUT1 (Nomura et al., 2010; Gurney et al., 2012). Furthermore, Cana blocked glucose influx-mediated β-catenin activation (Hung et al., 2019).

## 5 Conclusion

Canagliflozin provides a neuroprotective mechanism *via* PTEN/PDK/PPAR-γ signaling pathways in VPA-induced autism in rats. The current study confirmed that the protective effect of Cana against the induction of autism in rats with valproic acid involved significant ameliorating effect on the canonical Wnt/β-catenin pathway. This effect was reflected in improving the major core behaviors characterized for autism, enhancing sociability and social preference, inhibiting stereotypic behaviors, and decreasing hyperlocomotion activity with significant improvement of histopathological features of the brain.

## Data Availability

The original contributions presented in the study are included in the article/Supplementary Material; further inquiries can be directed to the corresponding author.
